# Racial and Ethnic Disparities in Second Primary Lung Cancer After Breast Radiotherapy: A SEER Cohort Analysis (2000–2022)

**DOI:** 10.3390/cancers18040635

**Published:** 2026-02-15

**Authors:** Fares A. Qtaishat, Mohammad Hamad, Adham Musa, Theeb Natsheh, Othman Al-Barghouthi, Basil A. Abusalameh, Anas A. Younis, Hamzeh Al-Qarallah, Sara Qutaishat, Matthew P. Banegas, H. Irene Su, Winta T. Mehtsun, Tala Al-Rousan

**Affiliations:** 1 School of Medicine, University of Jordan, Amman 11942, Jordan; far0223442@ju.edu.jo (F.A.Q.); mhm0214880@ju.edu.jo (M.H.); bas0233380@ju.edu.jo (B.A.A.); ans0229434@ju.edu.jo (A.A.Y.); hmz0200978@ju.edu.jo (H.A.-Q.); 2 College of Literature, Science, and the Arts, University of Michigan, Ann Arbor, MI 48109, USA; adhamm@umich.edu; 3 Faculty of Medicine, Jordan University of Science and Technology, Irbid 22110, Jordan; theeb2001@gmail.com (T.N.); othmanalbarghouthi@gmail.com (O.A.-B.); 4 Department of Internal Medicine, Medstar Union Memorial Hospital, Baltimore, MD 21218, USA; sara.a.qutaishat@medstar.net; 5 Department of Radiation Medicine and Applied Sciences, School of Medicine, University of California San Diego, La Jolla, CA 92093, USA; mbanegas@health.ucsd.edu; 6 Department of Obstetrics, Gynecology, and Reproductive Sciences, University of California San Diego, La Jolla, CA 92093, USA; hisu@health.ucsd.edu; 7 Division of Surgical Oncology, Department of Surgery, UC San Diego School of Medicine, La Jolla, CA 92093, USA; 8 Herbert Wertheim School of Public Health, University of California San Diego, La Jolla, CA 92093, USA

**Keywords:** breast cancer, adjuvant radiotherapy, second primary lung cancer, racial disparities, ethnic disparities, SEER database, cancer survivorship, marital status, survival outcomes, health disparities

## Abstract

Background: Adjuvant radiation therapy for breast cancer improves survival but may expose thoracic organs to low-dose radiation, increasing the risk of second primary lung cancer (SPLC). Racial and ethnic disparities and social factors influencing SPLC risk remain underexplored. Objectives: We quantified racial and ethnic differences in SPLC incidence and survival among radiotherapy-treated breast cancer survivors and assessed the potential protective role of marital status. Methods: Using SEER-17 (2000–2022), we identified patients with first primary breast cancer receiving radiotherapy, excluding those who died within two months. Standardized incidence ratios (SIRs) assessed observed versus expected SPLC cases by race and ethnicity and marital status, and five-year overall survival (OS) after SPLC and mean age at death were calculated. Racial categories included White, Black, Asian or Pacific Islander (API), and American Indian or Alaska Native (AI/AN); ethnicity was categorized as Hispanic or non-Hispanic. Results: Among 558,493 patients, 6674 developed SPLC (1.19%). Risk varied significantly by race (*p* < 0.05). AI/AN patients had the highest overall risk (SIR 1.82), particularly 12–59 months and ≥120 months post-treatment. Black (SIR 1.21) and API (SIR 1.23) survivors had sustained elevated risk, while White survivors showed no overall increase (SIR 0.96) and Hispanic survivors had lower risk (SIR 0.72). Married individuals had 12% lower SPLC incidence (SIR 0.88). Five-year overall survival after SPLC was 28.0%, with significant variation by race and ethnicity (*p* = 0.002). API (32.2%) and AI/AN (32.5%) patients had the highest survival, followed by White (28.0%) and Black patients (25.6%). Married patients had higher five-year survival (31.8% vs. 25.0%) and older mean age at death (64.3 vs. 48.6 years) compared to unmarried patients. Conclusions: SPLC risk and prognosis after breast radiotherapy differ by race, ethnicity, and marital status. These findings highlight the importance of context-aware survivorship counseling and support the consideration of personalized lung cancer screening for breast cancer survivors.

## Simple Summary

Radiation therapy is an important part of breast cancer treatment and helps many patients live longer. However, it can also expose nearby organs, such as the lungs, to low levels of radiation, which may increase the chance of developing lung cancer later in life. Not all breast cancer survivors face the same level of risk, and social factors may also influence outcomes. In this study, we examined a large U.S. cancer database to understand whether the risk of developing lung cancer after breast radiation differs by race, ethnicity, and marital status, as well as how these factors affect survival. We found that some racial groups and unmarried patients had higher risks and worse outcomes, while others had lower risk and better survival. These findings may help researchers and clinicians improve long-term follow-up care and design more personalized lung cancer screening strategies for breast cancer survivors.

## 1. Introduction

Breast cancer is the most common cancer among women in the United States and represents a significant public health burden [1]. Globally, the burden of breast cancer is expected to rise substantially, with projections estimating a 38% increase in incidence and a 68% increase in annual mortality by 2050, according to the International Agency for Research on Cancer (IARC), a specialized agency of the World Health Organization (WHO) [2,3]. The increased detection of early-stage breast cancer has resulted in a higher rate of breast-conserving surgeries followed by adjuvant radiotherapy (RT), now a standard component of multimodal treatment [4,5,6,7]. While advances in early detection and management have improved survival, treatment-related secondary malignancies have become increasingly recognized.

Although RT has significantly improved tumor control and survival outcomes, it also exposes adjacent thoracic structures to low-dose ionizing radiation, an important and established factor in the development of SPLC, especially among long-term survivors [8,9,10,11,12,13]. Previous studies have shown that breast cancer patients treated with RT may have up to double the risk of developing SPLC within ten years of treatment [9,10,11,12,13,14]. Additionally, recent evidence suggests that low-to-moderate radiation doses may be more carcinogenic than previously thought, and modern RT techniques have altered lung exposure patterns in clinically relevant ways. Specifically, intensity-modulated radiotherapy (IMRT) increases the volume of lung tissue exposed to low-dose radiation due to greater beam modulation, potentially elevating SPLC risk, whereas proton therapy reduces integral lung dose and may lower this risk, although long-term data remain limited [15,16,17]. Consequently, studies with extended follow-up beyond 10 years are needed to better define SPLC risk associated with contemporary RT techniques.

Beyond biological and treatment-related factors, social determinants of health (SDOHs), including socioeconomic status, insurance coverage, race and ethnicity, education, and neighborhood environment, are key drivers of cancer outcomes [18]. Among breast cancer survivors, inequities in access to care, health behaviors such as smoking, and continuity of follow-up contribute to disparities in both primary outcomes and long-term risks, including secondary lung cancer [19]. These factors may modify radiation-associated risk by shaping baseline susceptibility and post-treatment surveillance: in a large prospective cohort, ever-smokers had more than a threefold higher risk of second primary lung cancer than never-smokers (adjusted HR ~3.5), with risk increasing by ~24% per 10 pack-years, and nearly 80% of affected survivors did not meet current lung cancer screening criteria [20]. Understanding how SDOH intersect with cancer therapy and survivorship is essential to contextualizing observed disparities in secondary lung cancer incidence.

Research examining racial and ethnic disparities in second primary lung cancer following radiation exposure remains limited, despite consistent evidence that race and ethnicity modify cancer incidence, treatment delivery, survivorship care, and outcomes across oncology. The role of marital status in SPLC is even less well characterized; however, prior cancer research suggests that marital status may modify access to social support, adherence to post-treatment surveillance, and timeliness of medical follow-up, thereby potentially influencing SPLC detection and survival rather than risk alone. Given these gaps, a clearer understanding of how race, ethnicity, and marital status act as modifiers of SPLC risk and outcomes after radiotherapy is critically needed [21,22].

The utility of population-based cohort studies in evaluating long-term medical outcomes has been increasingly recognized. For instance, recent research regarding the risk of depression following cataract surgery demonstrated how large-scale longitudinal data can uncover complex psychological outcomes linked to sensory interventions [23]. Similarly, our study utilizes a population-based approach to stratify long-term risks, ensuring that the latency intervals analyzed capture a comprehensive view of patient outcomes over time.

This study addresses these gaps by quantifying the incidence and survival outcomes of SPLC among breast cancer survivors treated with radiotherapy, with a specific focus on racial and ethnic disparities and the modifying effect of marital status. Using population-based data from the Surveillance, Epidemiology, and End Results (SEER) database, this analysis provides contemporary estimates of SPLC risk in the setting of modern breast cancer management and highlights the importance of incorporating demographic and social determinants into survivorship planning and lung cancer screening strategies.

## 2. Methods

### 2.1. Data Source

Patients diagnosed with an SPLC, following prior diagnosis of breast cancer treated with radiation therapy, from 1 January 2000 to 31 December 2022 were collected from the SEER database using SEER* Stat software version 9.0.42 (www.seer.cancer.gov; accessed on 20 June 2025). Because SEER data are publicly available and de-identified, institutional review board (IRB) approval and informed consent were not required for this study.

### 2.2. Population

The population of this study is the SEER-17 population, which represents approximately one quarter of the United States population. The data collected and used are representative of a substantial portion, almost 26.5% of the American population, which supports the generalizability of the results (https://seer.cancer.gov/registries/; accessed on 20 June 2025).

### 2.3. Patient Selection

We included patients from the SEER database who met the following inclusion criteria: (1) diagnosed with primary breast cancer, using Site Recode ICD-O-3/WHO 2008 as “Breast” (ICD-O codes: 500-509); (2) received radiation therapy; (3) developed an SPLC (defined by SEER criteria for second primary malignancies (https://training.seer.cancer.gov/arc_neoplasms/; accessed on 20 June 2025)); (4) and had a minimum latency exclusion period of 2 months to avoid synchronous malignancies. Patients meeting the following criteria were excluded: prior cancer diagnoses, using the SEER filter for “First Primary Only” as Breast Cancer (Sequence Number = 0 or 1); incomplete follow-up; or missing racial information. Race was determined based on the “Race record” variable, which included White, Black, American Indian or Alaska Native (AI/AN), and Asian or Pacific Islander (API). Ethnicity was determined based on “race and ethnicity” variable, which was classified into Hispanic and non-Hispanic. Patients with missing or unknown race or ethnicity were excluded from primary comparative analyses.

### 2.4. Outcome Definition

The primary outcome was the development of second primary lung cancer (SPLC), defined as follows: (1) A new malignancy diagnosed ≥2 months after the initial breast cancer diagnosis to exclude synchronous cancers. (2) SPLCs were identified based on SEER definitions of primary tumor sites and behavior codes, excluding recurrences and metastases. Patients were followed until death or end of follow-up period.

### 2.5. Covariates

We collected clinicopathological data for each patient, including the following variables: Patient ID, age, gender, race, ethnicity, marital-status, histological type, radiation therapy types, chemotherapy, survival duration in months, tumor size, stage at diagnosis of primary tumor, metastasis at diagnosis, and laterality.

### 2.6. Statistical Analysis

The Multiple Primary Standardized Incidence Ratio (MP-SIR) session in SEER*Stat software version 8.4.4 and SPSS v.27 was used for all analyses. Standardized incidence ratios (SIRs) were calculated as the ratio of observed SPLC cases in the study cohort to the expected cases in the general population. Expected cases were calculated using age-, sex-, race-, and calendar year-specific incidence rates from the SEER reference population. Survival analysis was performed, where overall survival (OS) was estimated using Kaplan–Meier survival analysis. Survival differences among patient groups were assessed using log-rank tests. To account for potential confounding factors, multivariable Cox proportional hazards regression models were used. The multivariable Cox regression models were adjusted for age, sex, race, ethnicity, marital status, tumor size, stage at diagnosis, treatment modalities (radiation and chemotherapy), and tumor laterality. A *p*-value of <0.05 was considered significant. For subgroup analysis, SIRs were further stratified by race, ethnicity, and marital status. All statistical analyses were conducted using IBM SPSS Statistics version 27.0.

## 3. Results

### 3.1. Cohort Profile

This analysis included 6674 breast cancer survivors who received radiotherapy between 2000 and 2022 and were later diagnosed with second primary lung cancer (SPLC). On average, SPLC developed 7.01 years after the initial breast cancer diagnosis (SD 5.00), with latency ranging from 0 to 22 years. At diagnosis, the average age was 71.17 years (SD 9.90), and patients’ ages ranged from 32 to 92. Most of the cohort was female (99.6%). In terms of race, 85.4% of the patients were White (n = 5700), followed by 9.1% Black (n = 605), 5.1% Asian or Pacific Islander (n = 340), and 0.4% American Indian or Alaska Native (n = 29). Most patients (93.8%) were non-Hispanic, and 43.4% were married at the time they were first diagnosed with breast cancer. The location of the original breast tumor was almost evenly split, with 50.7% on the left side and 49.1% on the right. However, when SPLC developed, it occurred more often in the right lung (54.3%) than in the left (40.1%). The most common radiation type was beam radiation (n = 6256), accounting for (94.3%) of the total sample, while radioisotopes were the least common, with only four cases in total. (Table 1).

### 3.2. Histopathology

**Figure 1:**
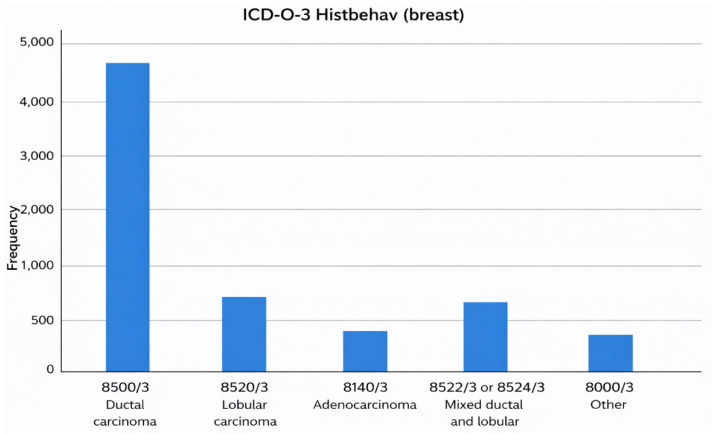
Histologic distribution of primary breast tumors (ICD-O-3 codes).

The histopathological types of primary breast cancer in the cohort varied, with the most common being invasive ductal carcinoma (74.0%, n = 4942). Mixed ductal and lobular carcinoma accounted for 10.0% of cases (n = 665), followed by lobular carcinoma at 7.7% (n = 516) and adenocarcinoma at 4.9% (n = 328). Other histologic subtypes comprised 3.3% of cases (n = 223) (Figure 1).

**Figure 2:**
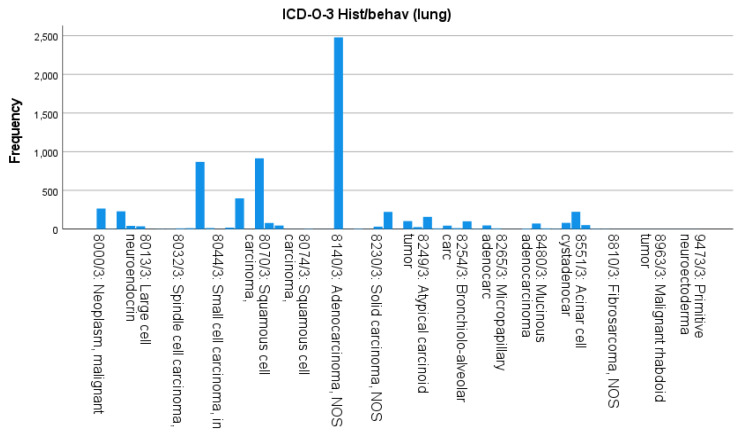
Histologic distribution of second primary lung cancers (ICD-O-3 codes).

In terms of SPLC histology, adenocarcinoma NOS (8140/3) was the most diagnosed type, accounting for 37.5% (n = 2500). This was followed by squamous-cell carcinoma NOS (8070/3) at 13.9% (n = 930), combined small-cell carcinoma variants (8044/3 and 8041/3) at 19.3% (n = 1288), solid carcinoma NOS (8230/3) at 4.0% (n = 268), and atypical carcinoid tumor (8249/3) at 3.4% (n = 225). All remaining subtypes together represented 21.9% of the SPLC cases (Figure 2).

### 3.3. Standardized Incidence Ratios (SIRs)

**Figure 3:**
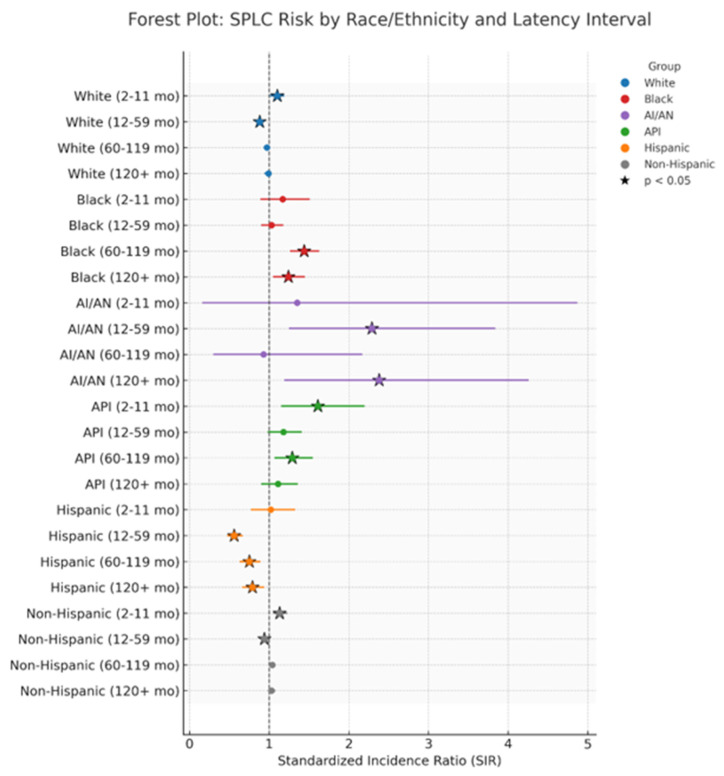
SPLC risk and latency interval according to race and ethnicity.

**Figure 4:**
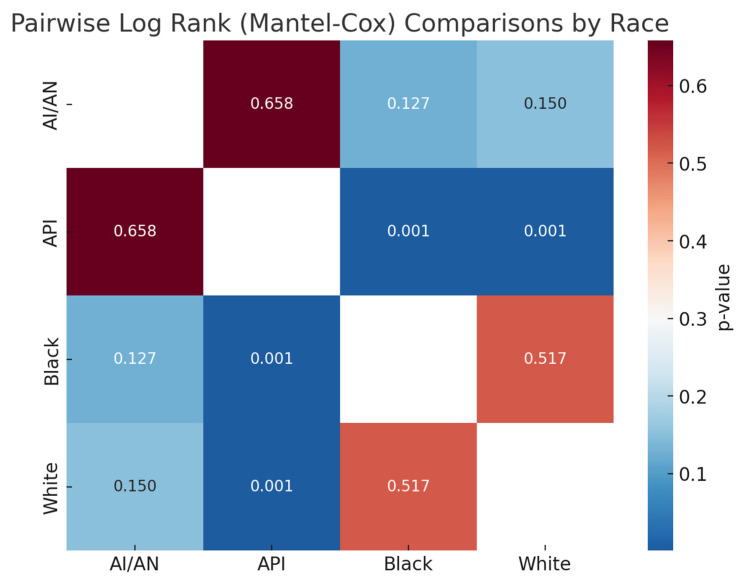
Pairwise log rank (Mantel–Cox) comparisons by race.

SIRs varied based on race and latency period. In the early phase (2–11 months), elevated SIRs were seen in Whites (1.11; 95% CI: 1.01–1.19), Blacks (1.47; 1.09–1.51), Asian/Pacific Islanders (1.61; 1.15–2.22), and American Indian/Alaska Natives (1.35; 0.16–4.87), though the latter was not statistically significant. Among White patients, risk decreased in the 12–59-month window (0.88; 0.80–0.92) and overall (0.96; 0.93–0.98). In contrast, Black patients experienced elevated risk across later periods—60–119 months (1.44; 1.26–1.63), ≥120 months (1.24; 1.05–1.45), and overall (1.21; 1.12–1.31). Asian/Pacific Islanders showed a consistently elevated overall risk (1.23; 1.11–1.36). Meanwhile, American Indian/Alaska Natives had significantly high SIRs at 12–59 months (2.29; 1.32–3.74), ≥120 months (2.38; 1.29–4.38), and overall (1.82; 1.24–2.57) (Table 2, Figure 3 and Figure 4).

**Table 2:** Standardized incidence ratios of second primary lung cancer by race and latency interval.

Among Races				
Duration	Observed	Expected	SIR	CI
White				
2–11 Months	579	527.37	1.1 #	1.01–1.19
12–59 Months	1985	2243.39	0.88 #	0.85–0.92
60–119 Months	1957	2018.48	0.97	0.93–1.01
120+ Months	1732	1756.82	0.99	0.94–1.03
Total	6253	6546	0.96 #	0.93–0.98
Black				
2–11 Months	59	50.36	1.17	0.89–1.51
12–59 Months	207	200.81	1.03	0.9–1.18
60–119 Months	235	163.44	1.44 #	1.26–1.63
120+ Months	155	125.5	1.24 #	1.05–1.45
Total	656	540.11	1.21 #	1.12–1.31
American Indian/Alaska Native				
2–11 Months	2	1.48	1.35	0.16–4.87
12–59 Months	14	6.12	2.29 #	1.25–3.84
60–119 Months	5	5.39	0.93	0.3–2.17
120+ Months	11	4.63	2.38 #	1.19–4.26
Total	32	17.61	1.82 #	1.24–2.57
Asian or Pacific Islander				
2–11 Months	39	24.21	1.61 #	1.15–2.2
12–59 Months	120	101.47	1.18	0.98–1.41
60–119 Months	119	92.03	1.29 #	1.07–1.55
120+ Months	95	85.28	1.11	0.9–1.36
Total	373	302.99	1.23 #	1.11–1.36
Unknown				
2–11 Months	<5	2.02	0.5	0.01–2.76
12–59 Months	<5	7.57	0.13 #	0–0.74
60–119 Months	0	5.74	0 #	0–0.64
120+ Months	0	4.4	0 #	0–0.84
Total	<5	19.73	0.10 #	0.01–0.37

#: *p*-value less than 0.05. Cells with fewer than five cases are suppressed to protect patient confidentiality, in accordance with SEER reporting guidelines.

Hispanic patients had consistently lower risk of developing SPLC. Their overall SIR was 0.72 (0.65–0.79), with further reductions during 12–59 months (0.56; 0.47–0.67), 60–119 months (0.75; 0.63–0.89), and ≥120 months (0.79; 0.66–0.94). By comparison, non-Hispanic patients showed a brief spike in early risk (2–11 months: 1.13; 1.05–1.23), followed by a small decrease at 12–59 months (0.94; 0.90–0.98), resulting in an overall neutral risk (1.01; 0.99–1.04) (Table 3, Figure 3).

Marital status also showed associations with SPLC risk. Married individuals had a lower overall risk (SIR 0.88; 0.85–0.91), with statistically significant reductions at 12–59 months (0.82; 0.77–0.87), 60–119 months (0.91; 0.86–0.96), and beyond 120 months (0.89; 0.84–0.94). In contrast, those who were unmarried had a higher risk during the 2–11-month interval (1.12; 1.04–1.21), a decreased risk at 12–59 months (0.91; 0.87–0.95), and a neutral overall risk (0.99; 0.96–1.01) (Table 4).

**Table 3:** Standardized incidence ratios by ethnicity and latency interval.

Ethnicity				
Duration	Observed	Expected	SIR	CI
Hispanic				
2–11 Months	55	53.83	1.02	0.77–1.33
12–59 Months	125	221.33	0.56 #	0.47–0.67
60–119 Months	144	191.52	0.75 #	0.63–0.89
120+ Months	127	161.58	0.79 #	0.66–0.94
Total	451	628.26	0.72 #	0.65–0.79
Non-Hispanic				
2–11 Months	624	550.24	1.13 #	1.05–1.23
12–59 Months	2202	2332.61	0.94 #	0.9–0.98
60–119 Months	2172	2088.90	1.04	1–1.08
120+ Months	1866	1811.24	1.03	0.98–1.08
Total	6864	6782.99	1.01	0.99–1.04
Unknown				
2–11 Months	<5	1.37	0.73	0.02–4.06
12–59 Months	0	5.42	0.00 #	0–0.68
60–119 Months	0	4.64	0.00 #	0–0.79
120+ Months	0	3.81	0.00 #	0–0.97
Total	<5	15.25	0.07 #	0–0.37

#: *p*-value less than 0.05. Cells with fewer than five cases are suppressed to protect patient confidentiality, in accordance with SEER reporting guidelines.

**Table 4:** Standardized incidence ratios by marital status and latency interval.

Marital Status				
Duration	Observed	Expected	SIR	CI
Married				
2–11 Months	321	331.45	0.97	0.87–1.08
12–59 Months	1180	1443.96	0.82 #	0.77–0.87
60–119 Months	1236	1362.23	0.91 #	0.86–0.96
120+ Months	1145	1284.98	0.89 #	0.84–0.94
Total	3882	4422.63	0.88 #	0.85–0.91
Unmarried				
2–11 Months	680	605.44	1.12 #	1.04–1.21
12–59 Months	2327	2559.36	0.91 #	0.87–0.95
60–119 Months	2316	2285.07	1.01	0.97–1.06
120+ Months	1993	1976.63	1.01	0.96–1.05
Total	7316	7426.50	0.99	0.96–1.01

#: *p*-value less than 0.05.

### 3.4. Survival Analysis

**Figure 5:**
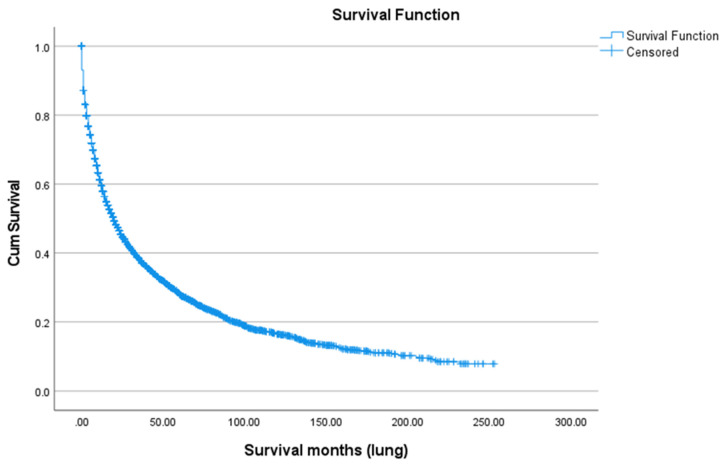
Overall survival among SPLC patients.

**Figure 6:**
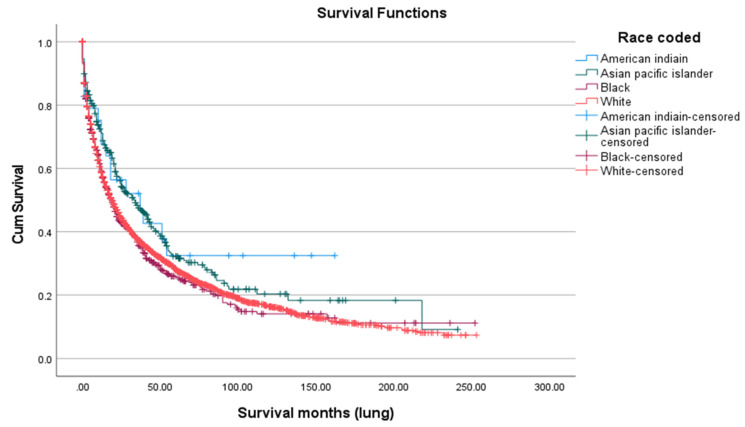
Survival according to race.

**Figure 7:**
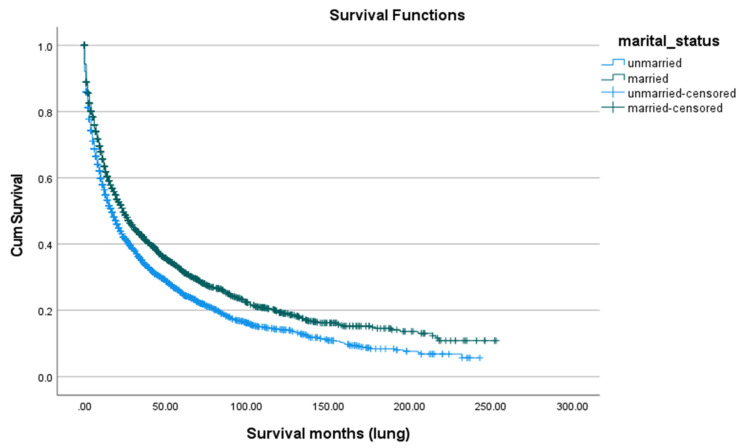
Survival according to marital status.

**Figure 8:**
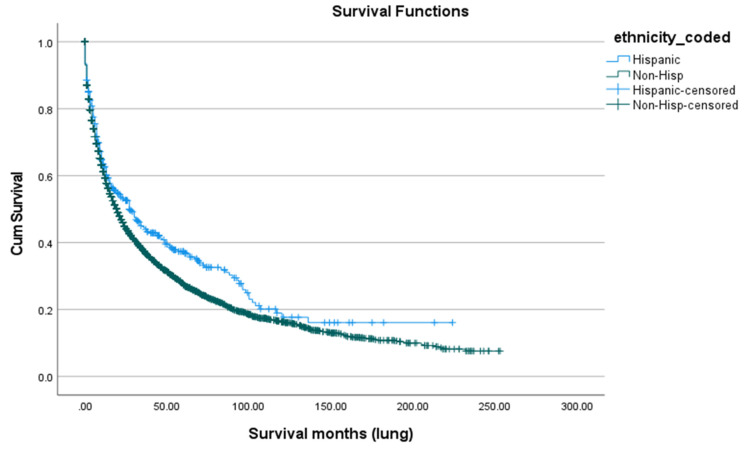
Survival according to ethnicity.

**Figure 9:**
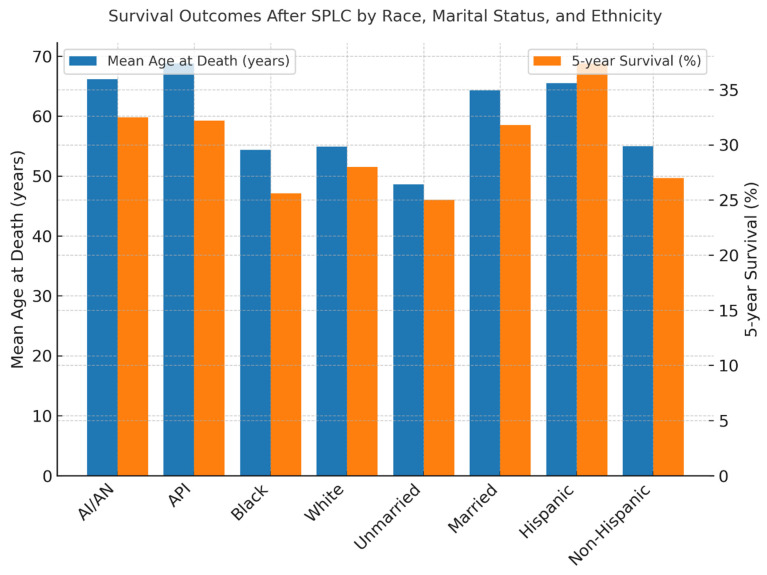
Combined groups’ survival outcomes.

Survival after SPLC diagnosis was generally poor, with a five-year overall survival rate of 28.0% (Figure 5). There were notable differences by race and ethnicity. Asian/Pacific Islanders had the highest five-year survival (32.2%) and the highest mean age at death (68.82 years), followed by American Indian/Alaska Natives (32.5%; 66.17 years), Whites (28.0%; 54.94 years), and Blacks (25.6%; 54.39 years), with a statistically significant difference (*p* < 0.01) (Figure 6). Marital status also played a role: married patients lived longer (mean age at death: 64.31 years) and had a higher five-year survival rate (31.8%) compared to unmarried individuals (48.64 years; 25.0%, *p* < 0.01) (Figure 7). Hispanic patients also fared better than non-Hispanics, with a mean survival age of 65.48 years and a five-year survival of 37.4%, compared to 55.0 years and 27.0% in non-Hispanics (*p* = 0.005) (Table 5, Figure 8 and Figure 9).

**Table 5:** Overall and subgroup survival following second primary lung cancer.

Characteristic	Mean Age of Survival	Confidence Interval (95%)	5 Year Survival	*p*-Value
Race				
American	66.165	38.89–93.439	32.5%	<0.01
Asian	68.819	56.76–80.88	32.2%	<0.01
Black	54.391	45.67–63.11	25.6%	<0.01
White	54.943	52.3–57.59	28%	<0.01
Marital Status				
unmarried	48.638	45.70–51.58	25%	0.000
married	64.306	60.26–68.36	31.80%	0.000
Ethnicity				
Hispanic	65.480	55.09–75.88	37.40%	<0.01
Non-Hispanic	54.969	52.44–57.50	27%	<0.01

### 3.5. Cox Proportional Hazards Regression

The analysis identified several significant demographic and clinical predictors of mortality. Marital status was a key factor, with unmarried patients showing a higher risk of death than married patients (HR 1.14, 95% CI 1.07–1.21; *p* < 0.01). Hispanic ethnicity was associated with a lower hazard compared with non-Hispanic ethnicity (HR 0.84, 95% CI 0.74–0.96; *p* < 0.01). Overall stage was not significant (*p* = 0.27); however, Stage IV predicted increased mortality (HR 1.37, 95% CI 1.01–1.85; *p* = 0.04). Breast histology was significant in outcomes (overall *p* < 0.01), with ductal, lobular, adenocarcinoma, and mixed ductal/lobular subtypes demonstrating reduced hazards (HR range 0.68–0.77; all *p* ≤ 0.01). Race showed a borderline statistically significant association with mortality (overall *p* = 0.05), largely driven by the lower risk observed among Asian/Pacific Islander patients (HR 0.86, 95% CI 0.74–0.99; *p* = 0.03). Mortality increased with age (HR 1.02 per year, 95% CI 1.02–1.03; *p* < 0.01), while tumor side (*p* = 0.47) and sex (*p* = 0.87) were not significantly associated with survival. Radiation type was not significantly associated with mortality (overall *p* = 0.15). Across radiation categories, there were no statistically significant differences in HR (beam radiation: HR 1.12, *p* = 0.80; combination: HR 1.56, *p* = 0.55; radiation NOS: HR 1.32, *p* = 0.70; radioactive implants: HR 1.02, *p* = 0.98) (Table 6).

## 4. Discussion

In this population-based study using SEER data, we investigated the incidence and survival outcomes of SPLC following breast cancer radiotherapy, with a focus on racial/ethnic disparities and the influence of marital status. Our study demonstrated that the incidence of SPLC following breast cancer radiotherapy was elevated most significantly among AI/ANs, who experienced risks more than double the expected rate. The incidence was also higher among Black individuals, as well as APIs. Additionally, we observed consistently low SPLC risks among married patients, suggesting a potential protective effect.

The results on the incidence of SPLC align with the findings of the current literature, which report an elevated long-term risk of SPLC after breast radiotherapy. The increased risk of SPLC following breast radiotherapy observed in Black, API, and AI/AN patients may suggest a persistent biological vulnerability or environmental exposures that are not mitigated by modern radiotherapy protocols. Nevertheless, the risk is thought to be lower than previously published, with an incidence of 1.19% in our study compared to 1.74% in [24]. While the primary lung cancer incidence rate in Black individuals has been consistently higher than that in Whites [25], data showed that AI/ANs had a lower incidence of primary lung cancer than Whites [26]. Our study reveals that the risk in AI/AN populations is more pronounced than previously reported. This is a significant contribution, as few studies have specifically examined this group in the context of radiation-induced cancers.

Our analysis showed that married patients had a lower likelihood of SPLC over both 5-year and 10-year periods, which is considered the most important regarding radiation-related malignancies. While unmarried patients had a higher likelihood of SPLC during the first year, this likelihood decreased over time. This suggests that social support has a vital role to play in reducing the risk of having lung cancer, as it helps patients to recognize respiratory symptoms as they arise and may also contribute to a reduction in smoking rates [27].

The median OS for patients with SPLC in our study differed significantly among racial groups, with API having the longest OS at 68.8 months, followed by AI/AN and White individuals; Black patients showed the shortest OS at 54.4 months. The five-year survival also varied between racial groups, being highest among Hispanics and lowest among Black individuals. This is likely due to multiple variables on different levels, including patient-level factors such as socioeconomic barriers, cultural stigma, mistrust, and insurance limitations, as well as physician-level variables such as implicit bias, stereotyping, and inconsistent treatment recommendations. Finally, system-level factors play a crucial role and include, but are not limited to, unequal access to high-quality care, underrepresentation in trials, and delayed treatment [28].

Our study found that Hispanic breast cancer survivors had a lower risk of SPLC and better five-year survival compared with non-Hispanic patients. This pattern is consistent with the well-described lower baseline incidence of lung cancer among Hispanic populations, a trend largely attributed to lower smoking prevalence, cultural factors, and differences in environmental exposures. These established population patterns likely contribute to the reduced SPLC risk observed in our cohort. Further research is needed to clarify how these factors interact with prior radiation exposure and to ensure appropriate risk-based follow-up. [29,30,31]. It is important to note that, some other factors may differ among the Hispanic ethnic group, such as country of origin, but such information was not available.

Although the cohort had a mean age of 71.17 years at SPLC diagnosis, the mean age at death following SPLC diagnosis was lower and varied across racial and ethnic groups. This occurs because age at death is calculated only among individuals who died during the follow-up period, whereas a substantial proportion of patients remained alive at the end of follow-up and were therefore censored.

For married individuals, survival outcomes were superior to those of unmarried patients. Likely due to psychosocial, economic, and environmental factors, having a partner or spouse is associated with a healthier lifestyle, a greater chance of detecting the disease at an earlier stage, and a higher likelihood of opting for active treatment [32].

Multiple risk factors have been described to affect the incidence of SPLC following breast radiotherapy, including smoking [33], dose [34], and type of radiation (proton vs. photon therapies) [35]. Findings from our study suggest increased risk in certain racial groups and people who are unmarried, raising the need to design a risk stratification tool to include the abovementioned risk factors and better predict the risk of SPLC associated with breast radiotherapy. Better screening and surveillance should also be implemented based on such a tool.

Beyond marital status, quality of life (QoL) and social factors are increasingly recognized as critical determinants of long-term survivorship in patients with severe, life-altering diseases. Evidence from other cohorts, such as stroke survivors assessed using the Vietnamese version of the Stroke Impact Scale 3.0, highlights how psychosocial support, functional independence, and social engagement significantly influence recovery, well-being, and adherence to care [36]. By analogy, the protective effect observed among married patients in our cohort may reflect not only emotional support but also enhanced social resources, earlier detection of health issues, and more effective engagement with healthcare services. Incorporating assessments of QoL and social determinants into future studies of SPLC survivors could provide a more comprehensive understanding of survivorship and inform interventions aimed at mitigating the adverse impact of cancer and its treatment on daily life.

This study is limited by its reliance on retrospective data and the SEER database. As a registry-based dataset, SEER lacks important clinical variables such as smoking status, radiation dose, treatment details, and key comorbidities, which restricts the ability to adjust for major confounders. Consequently, our findings should be interpreted with caution due to the complexity of the interaction between such factors. SEER also does not capture some essential socioeconomic indicators, limiting the robustness of statistical adjustments for social determinants of health. Additionally, SEER covers approximately half of the U.S. population, which may limit the generalizability of our findings to non-SEER regions. Follow-up and latency definitions are based on SEER multiple primary rules, which may result in shorter follow-up windows than ideal for capturing radiation-associated SPLCs. Changes in radiation therapies over the study period, for example, the shift from 2D to 3D conformal methods, have improved treatment quality and reduced errors; however, this temporal heterogeneity may bias observed associations toward the null. Future research using prospective cohorts with comprehensive clinical and socioeconomic information is needed to better evaluate the roles of smoking, radiation dose, comorbidities, and marital status in SPLC risk after breast radiotherapy. Also, looking at the effect of radiation among breast cancer patients would give extra insight into the subject. Moreover, further studies are needed to better characterize the biological and clinical significance of SPLCs occurring shortly after breast cancer diagnosis and radiotherapy.

## 5. Conclusions

This population-based cohort study demonstrates that the risk and survival outcomes of SPLC after breast cancer radiotherapy vary significantly by race, ethnicity, and marital status. These findings highlight the need to integrate sociodemographic factors into survivorship care and long-term surveillance strategies for breast cancer patients receiving radiotherapy. Future work should prioritize developing personalized risk-stratification models that incorporate race, social context, and treatment history to guide equitable lung cancer screening and follow-up.

## Figures and Tables

**Table 1:** Baseline characteristics of 6674 breast-cancer survivors who developed a second primary lung cancer.

Characteristics	Mean (SD)	Range (Min/Max)
Latency Period	7.01 (5.003)	22 (0/22)
Age	71.17 (9.90)	60 (32/92)
Characteristics	Frequency (n = 6674)	Percent
Race		
White	5700	85.4
Black	605	9.1
Asian/Pacific Islander	340	5.1
American Indian/Alaska Native	29	0.4
Sex		
Female	6646	99.6
Male	28	0.4
Ethnicity		
Non-Hispanic	6258	93.8
Hispanic	416	6.2
Marital status		
Married (including common law)	2899	43.4
Widowed	1730	25.9
Divorced	876	13.1
Single (never married)	782	11.7
Unknown	326	4.9
Separated	49	0.7
Unmarried or Domestic	12	0.2
Laterality (breast)		
Left—origin of primary	3386	50.7
Right—origin of primary	3274	49.1
Paired site, but no information	12	0.2
Only one side—side unspecified	2	0.0
Laterality (lung)		
Right—origin of primary	3626	54.3
Left—origin of primary	2675	40.1
Paired site, but no information	284	4.3
Bilateral, single primary	66	1.0
Only one side—side unspecified	17	0.3
Not a paired site	6	0.1

**Table 6:** Cox proportional hazard regression.

		*p*-Value	HR	95.0% CI	
				Lower	Upper
Marital status	Unmarried vs. Married *	0.00	1.14	1.07	1.21
Ethnicity	Hispanic vs. Non-Hispanic *	<0.01	0.84	0.74	0.96
Stage	Overall	0.27			
	Stage (1)	0.72	1.03	0.87	1.22
	Stage (2)	0.55	1.06	0.89	1.26
	Stage (3)	0.60	1.05	0.87	1.27
	Stage (4)	0.04	1.37	1.01	1.85
Breast Histology	Overall	<0.01			
	Ductal	<0.01	0.76	0.65	0.89
	Lobular	<0.01	0.72	0.60	0.87
	Adenocarcinoma	0.01	0.77	0.63	0.94
	Mixed Ductal/Lobular	0.00	0.68	0.57	0.81
Side	Overall	0.47			
	Same side	0.73	0.99	0.92	1.06
	Opposite side	0.22	0.95	0.88	1.03
Age	Mean Age	0.00	1.02	1.02	1.03
Sex	Female vs. Male *	0.87	0.96	0.60	1.55
Race	Overall	0.05			
	American Indian/Alaska Native	0.19	0.72	0.45	1.17
	Asian or Pacific Islander	0.03	0.86	0.74	0.99
	Black	0.29	1.06	0.95	1.17
Radiation (RA)	Overall	0.15			
	Beam RA	0.80	1.20	0.30	4.79
	Combination of RA	0.55	1.56	0.37	6.66
	RA, (NOS)	0.70	1.32	0.32	5.41
	RA implants	0.98	1.02	0.25	4.10

*: reference value.

## Data Availability

Restrictions apply to the availability of these data. Data were obtained from the Surveillance, Epidemiology, and End Results (SEER) database and are available at https://seer.cancer.gov/data/access.html; accessed on 20 June 2025 with the permission of SEER.
